# Hypoxic preconditioning potentiates the trophic effects of mesenchymal stem cells on co-cultured human primary hepatocytes

**DOI:** 10.1186/s13287-015-0218-7

**Published:** 2015-12-01

**Authors:** Harry H. Qin, Céline Filippi, Song Sun, Sharon Lehec, Anil Dhawan, Robin D. Hughes

**Affiliations:** Dhawan Lab, Institute of Liver Studies, King’s College London and King’s College Hospital NHS Foundation Trust, London, SE5 9PJ UK; NIHR Biomedical Research Centre at Guy’s and St Thomas NHS Foundation Trust and King’s College London, London, SE1 9RT UK; Paediatric Liver, GI and Nutrition Centre, King’s College Hospital Denmark Hill, London, SE5 9RS UK

**Keywords:** Mesenchymal stem cell, Hepatocyte, Co-culture, Hypoxic preconditioning, Reactive oxygen species, Collagen, Cytokines, Apoptosis

## Abstract

**Introduction:**

Mesenchymal stem/stromal cells (MSCs) improve the metabolic function of co-cultured hepatocytes. The present study aimed to further enhance the trophic effects of co-culture with hepatocytes using hypoxic preconditioning (HPc) of the MSCs and also to investigate the underlying molecular mechanisms involved.

**Methods:**

Human adipose tissue-derived MSCs were subjected to hypoxia (2 % O_2_; HPc) or normoxia (20 % O_2_) for 24 h and then co-cultured with isolated human hepatocytes. Assays of metabolic function and apoptosis were performed to investigate the hepatotrophic and anti-apoptotic effects of co-culture. Indirect co-cultures and co-culture with MSC-conditioned medium investigated the role of paracrine factors in the hepatotrophic effects of co-culture. Reactive oxygen species (ROS) activity was antagonised with N-acetylcysteine to investigate whether HPc potentiated the effects of MSCs by intracellular ROS-dependent mechanisms. Tumour necrosis factor (TNF)-α, transforming growth factor (TGF)-β1, and extracellular collagen production was determined and CASP9 and BAX/BCL-2 signalling pathways analysed to investigate the role of soluble factors, extracellular matrix deposition, and apoptosis-associated gene signalling in the effects of co-culture.

**Results:**

HPc potentiated the hepatotrophic and anti-apoptotic effects of co-culture by ROS-dependent mechanisms. There was increased MSC TGF-β1 production, and enhanced MSC deposition of extracellular collagen, with reduced synthesis of TNF-α, as well as a downregulation of the expression of pro-apoptotic *CASP9*, *BAX*, *BID* and *BLK* genes and upregulated expression of anti-apoptotic *BCL-2* in hepatocytes.

**Conclusions:**

HPc potentiated the trophic and anti-apoptotic effects of MSCs on hepatocytes via mechanisms including intracellular ROS, autocrine TGF-β, extracellular collagen and caspase and BAX/BCL-2 signalling pathways.

**Electronic supplementary material:**

The online version of this article (doi:10.1186/s13287-015-0218-7) contains supplementary material, which is available to authorized users.

## Introduction

Hepatocyte transplantation has emerged as a promising alternative treatment for patients with end-stage liver disease, particularly those unsuitable for or without access to liver transplantation [1]. However, the limited availability of donor liver and the quality of cells isolated from marginal livers restricts the wider use of this technique. Once isolated and cultured in vitro, primary hepatocytes lose their proliferative potential and show a rapid phenotypic de-differentiation and metabolic regression in mono-culture [2]. Clinically feasible methods are required to preserve and improve quality and functionality of human hepatocytes during and after isolation.

Mesenchymal stem cells (MSCs) have been shown to have a supportive effect on hepatocytes in long-term co-culture in vitro [3]. MSCs maintain and improve co-cultured hepatocyte morphology and metabolic function by the synergistic effects of soluble factors, extracellular matrix (ECM), and cell–cell communication [4–7]. Our previous study demonstrated that co-culture with MSCs improved human hepatocyte viability and metabolic function in a heterotypic cell contact-dependent manner [8].

MSCs normally reside in a physiologically hypoxic niche, such as adipose tissue. Hypoxic preconditioning (HPc) has been used to protect MSCs from hypoxia/reoxygenation-induced apoptosis by stabilising the mitochondrial membrane potential, upregulating Bcl-2 and vascular endothelial growth factor (VEGF) signalling, and promoting the phosphorylation of mutagens-activated protein kinase/extracellular signal regulated kinase (MAPK/ERK) and Akt signalling pathways [9]. HPc significantly increases the expression of pro-survival and pro-angiogenic factors, such as hypoxia-inducible factor 1α, angiopoietin 1, VEGF and its receptor, erythropoietin, Bcl-2, and Bcl-xL, and also significantly decreases caspase-3-initiated cellular apoptosis in MSCs [10]. Beneficial therapeutic effects of transplanting HPc-MSCs have been found in experimental limb, cerebral, renal, and spinal cord ischaemia in animals. HPc can also rejuvenate aged adipose tissue-derived MSCs by upregulating the gene expression of pro-angiogenic factors, including VEGF, placental growth factor and hepatocyte growth factor [11].

The main aim of this study was to investigate whether HPc of MSCs could potentiate their trophic effects on co-cultured human primary hepatocytes. In addition, we aimed to investigate the critical role of reactive oxygen species (ROS) in MSC HPc, and the mechanisms underlying effects of MSC HPc in hepatocyte co-culture, including soluble factors, cell–cell contact, cell–matrix interactions, and hepatocyte pro-apoptotic and anti-apoptotic signalling.

## Methods

All reagents were purchased from Sigma-Aldrich (Poole, Dorset, UK) unless otherwise specified.

### Adipose tissue MSC hypoxic preconditioning

Human primary adipose tissue-derived MSCs were purchased from Invitrogen Ltd (Paisley, UK), and passages 6−8 (P6–P8) of MSCs were subcultured using a low-serum MSC expansion medium (Invitrogen). MSCs were plated on collagen-coated microplates (Fisher Scientific UK Ltd., Leicester, UK) at an optimised density of 20,000 viable cells per cm^2^. After 8 h culture, MSCs were subjected to HPc using a hypoxia incubator chamber (StemCell Technologies SARL, Sirocco, France) which was first purged of O_2_ by gassing with 95 % N_2_ plus 5 % CO_2_ (20 L/min for 3 min). MSC were incubated at 37 °C for an optimised time of 24 h in the hypoxic atmosphere (2 % O_2_). Normoxia-preconditioned (NPc) MSCs under 95 % air plus 5 % CO_2_ (20 % O_2_) were used as control.

### Hepatocyte mono-culture and co-culture

Human primary hepatocytes were isolated from donor liver tissues using a standard collagenase perfusion technique as previously reported [12]. The use of human liver tissues was approved by the Research Ethics Committee at King’s College Hospital, London, UK in accordance with the Human Tissues Act of 2004. All donors or their legal representatives volunteered to give informed consent in writing for research use of donor liver. Batches of hepatocytes with a viability of over 60 % on trypan blue exclusion were used for experiments.

For hepatocyte mono-culture, hepatocytes were plated onto collagen-coated microplates at an optimised density of 50,000 viable cells per cm^2^ in a chemically defined hepatocyte culture medium as previously described [12]. For hepatocyte co-culture, fresh hepatocytes were seeded at the optimised density on top of a MSC monolayer (MSC:hepatocyte = 1:2.5). Indirect co-culture was performed with MSCs plated on Transwell Permeable Supports (Corning Incorporated, Corning, NY, USA) which have culture inserts to separate the two types of cells, so that the contribution of soluble factors to the effects of co-culture could be determined by excluding ECM and cell–cell contact. Fresh hepatocytes were also cultured in co-culture conditioned medium to investigate whether paracrine mechanisms mediate synergistic effects of soluble factors, ECM and MSC–hepatocyte contact in co-culture. Culture medium was refreshed on every alternate day.

### Albumin and urea assays

A human albumin enzyme-linked immunosorbent assay (ELISA) quantitation kit (Bethyl Laboratories, Inc., Montgomery, TX, USA) and QuantiChrom™ urea assay kit (BioAssay Systems, Hayward, CA, USA) were used to quantitate free albumin and urea levels, respectively, as measures of hepatocyte function. Albumin secretion and urea synthesis of hepatocytes in mono- and co-culture were normalised to one million seeded viable hepatocytes.

### Effect of MSC co-culture on hepatocyte apoptosis and cell death

Caspase-cleaved cytokeratin 18 (CCK18), a marker of hepatocyte apoptosis, and total cytokeratin 18 (CK18), a marker of hepatocyte death, were quantitated using the M30 CytoDeath and M65 EpiDeath ELISA kits (PEVIVA AB, Bromma, Sweden), respectively. Levels of soluble CCK18 and CK18 released from hepatocytes in mono- and co-culture were normalised to one million seeded viable hepatocytes. The ratio of CCK18 to CK18 was also calculated to determine the relative mode of death for hepatocytes in vitro [13]. Staurosporine, a prototypical adenosine triphosphate (ATP)-competitive protein kinase inhibitor activating caspase-3 signalling [14], was used to induce apoptosis to investigate whether MSC co-culture specifically inhibited hepatocyte apoptosis via the caspase signalling pathway. Fresh hepatocyte mono-cultures were treated with 0 (blank control), 0.5, 1, 2.5, 5, and 10 μM staurosporine for 24 h to induce apoptosis and then subsequently co-cultured with MSCs for 24 h.

### Role of ROS in the effects of MSCs on hepatocytes

**Fig. 1:**
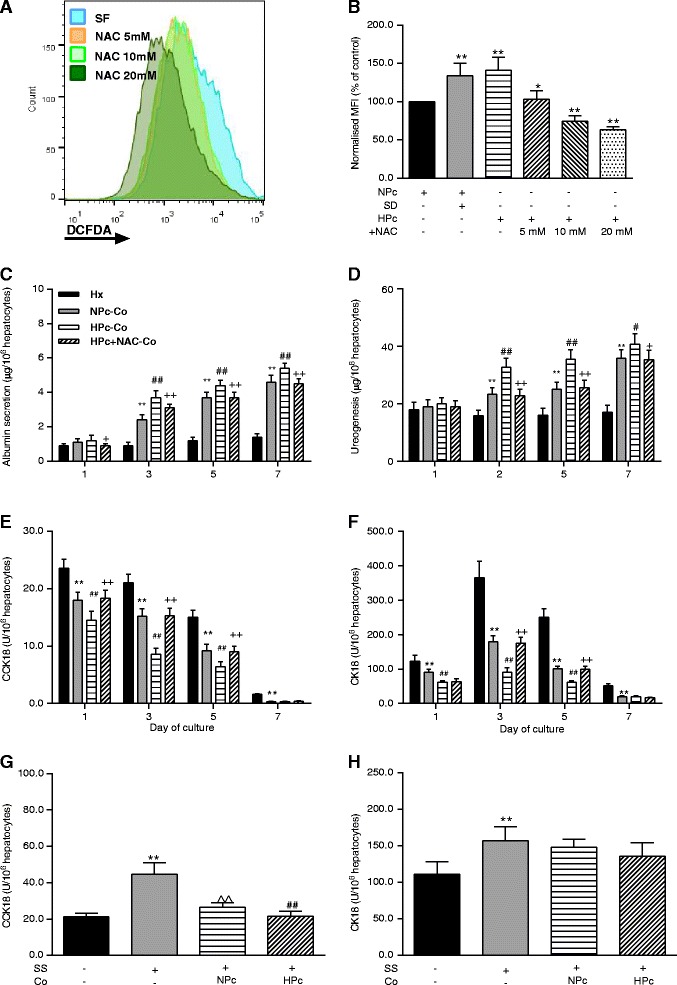
HPc potentiates the hepatotrophic and anti-apoptotic effects of co-culture with MSCs. **a** Representative histogram of DCFDA to measure intracellular ROS fluorescence intensity of serum-deprived, HPc-, and HPc + NAC-pretreated MSCs. **b** Bar chart of normalised MFI of intra-MSC ROS activity. Values are mean ± standard deviation (*n* = 6). *p < 0.05 and **p < 0.01, versus NPc-MSCs. **c** Albumin secretion and **d** urea synthesis of mono- and co-cultured hepatocytes with NPc- or HPc-MSCs, with or without NAC 10 mM. **e** Soluble CCK18 and **f** CK18 releases of mono- and co-cultured hepatocytes. **g** Staurosporine cytotoxicity assay of mono-cultured hepatocytes and resistance of co-cultured hepatocytes to 1 μM staurosporine-induced cellular apoptosis and **h** total death. Values are mean ± standard deviation (*n* = 6). **p < 0.01, versus hepatocyte mono-culture; ^##^ *p* <0.01, versus co-culture with NPc-MSCs; ^+^ *p* <0.05 and ^++^ *p* <0.01, versus co-culture with HPc-MSCs; ^^*p* <0.01, versus hepatocyte mono-culture treated with 1 μM staurosporine. *CCK18* Caspase-cleaved cytokeratin 18, *CK18* Cytokeratin 18, *Co* Co-culture; *DCFDA* Dichloro-dihydrofluorescein diacetate acetyl ester, *HPc* Hypoxia-preconditioned, *Hx* Hepatocyte mono-culture; *MFI* Median fluorescence intensity, *NAC* N-acetylcysteine, *NPc* Normoxia-preconditioned, *SD* Serum-deprived, *SF Serum-free*, *SS* Staurosporine

Hypoxia increases ROS activity in MSCs. Intracellular ROS activity was measured in MSCs using an 8-channel BD FACS Canto II flow meter (BD Biosciences, San Jose, CA, USA) with dichloro-dihydrofluorescein diacetate acetyl ester (Molecular Probes, Inc., Eugene, OR, USA) staining as previously described [15]. Median fluorescence intensities (MFIs) of HPc- and serum-deprived (positive control) MSCs were normalised to that of NPc-MSCs [16]. N-acetylcysteine (NAC), a potent scavenger of ROS, was used to block ROS to investigate whether increased ROS mediated the HPc-induced effects of co-culture of MSCs with hepatocytes. MSCs were pretreated with 0, 5, 10, and 20 mM NAC (PLIVA Pharma, Ltd., Hampshire, UK) for 24 h and then the MSCs were co-cultured with hepatocytes for 7 days. Intracellular ROS activity of NAC-pretreated, HPc-MSCs was quantitated prior to co-culture with hepatocytes (Fig. 1a).

### Involvement of tumour necrosis factor-α and transforming growth factor-β1 in the effects of MSCs on hepatocytes in co-culture

The supernatant levels of soluble tumour necrosis factor (TNF)-α and transforming growth factor (TGF)-β1 were determined during MSC and hepatocyte co-culture, including in the presence of NAC, using Quantikine® human TNF-α and TGF-β1 ELISA immunoassay kits (R&D Systems Europe, Ltd., Abingdon, UK). TNF-α and TGF-β1 neutralisation experiments with antibodies were performed to investigate the pro-apoptotic effect of TNF-α on mono-cultured hepatocytes and role of release of TGF-β1 from MSCs in the co-culture trophic effects. For this, HPc-induced potentiation together with 24-h incubation with 0 (blank control), 10, 25, 50 100, and 200 μg/mL TNF-α antibodies and 1, 2.5, 5, 10, and 20 μg/mL TGF-β1 antibodies (R&D Systems Europe Ltd.) was performed.

### Role of collagen in MSC and hepatocyte interaction

Collagen could be implicated in the trophic effects of co-culture with MSCs and HPc-induced potentiation. Cellular, free and extracellular collagen content of cell cultures were quantitated using the picro-sirius red colorimetric assay with the Sircol soluble collagen assay kit (Biocolor, Carrickfergus, UK). MSCs were pretreated with 0, 0.1, 0.5, 1, 2.5, 5, 10, and 20 mM N-(methylamino)-isobutyric acid, a competitive inhibitor of the neutral amino acid transport A system in collagen synthesis [17], for 24 h prior to 24-h co-culture.

### Expression of pro-apoptosis-associated and anti-apoptosis-associated genes during co-culture of MSC with hepatocytes

Total RNA samples were extracted using the Direct-zol RNA MiniPrep kit (Zymo Research Corporation, Irvine, CA, USA) and reversely transcribed into complementary DNA (cDNA) using the Omniscript Reverse Transcriptase kit (Qiagen, West Sussex, UK). cDNA samples were amplified using the TaqMan PreAmp master mix kit (Applied Biosystems, Life Technologies, Paisley, UK). Quantitative real-time polymerase chain reaction (qRT-PCR) assay was performed using the ABI PRISM 7000 sequence detection system (Applied Biosystems) with primers (Table 1) synthesised by Invitrogen for human gene expression assays of caspases (*CASP*) 3, 8, 9 and 14, Bcl-2-associated X protein 4 (*BAX*), *BCL-2*, BH3 interacting-domain death agonist (*BID*) and B lymphoid tyrosine kinase (*BLK*). Cycle threshold (Ct) was produced with an automatic threshold using the RQ Study Application (Applied Biosystems). The qPCR results were analysed using the semi-quantitative 2^-ΔΔCt^ method [18] and expressed as mRNA expression level relative to that of mono-cultured hepatocytes (control).

**Table 1:** qPCR primer identification

Gene symbol	Assay ID	Dye label
*B2M* (reference gene)	Hs00984230_m1	VIC
*CASP3*	Hs00234385_m1	FAM
*CASP8*	Hs01018151_m1	FAM
*CASP9*	Hs00154261_m1	FAM
*CASP14*	Hs00201637_m1	FAM
*BAX*	Hs00180269_m1	FAM
*BCL-2*	Hs00236808_s1	FAM
*BID*	Hs00609632_m1	FAM
*BLK*	Hs00176441_m1	FAM

*B2M* Beta-2 microglobulin, *BAX* BCL-2-associated X protein, *BCL-2* B-cell lymphoma 2, *BID* BH3 interacting-domain death agonist, *BLK* B lymphoid tyrosine kinase, *CASP* Caspases, *qPCR* Quantitative polymerase chain reaction

### Statistical analysis

All experiments were performed in duplicate and independently repeated in triplicate. GraphPad Prism 6 programme (GraphPad Software, Inc., La Jolla, CA, USA) was used for statistical analysis. All continuous data were expressed as mean ± standard deviation (SD), and the means were compared using the one-way repeated measures analysis of variance, the Fisher's least significant difference test, or two independent samples student *t*-test unless specified otherwise. A two-tailed *p*-value <0.05 was considered statistically significant.

## Results

### HPc of MSCs potentiates the effects of co-culture with hepatocytes via intracellular ROS-dependent mechanisms

HPc did not result in cellular detachment, necrosis, or morphological distortion of MSCs (data not shown). HPc and serum deprivation significantly increased MSC intracellular ROS activity as compared to NPc (both *p* < 0.01; Fig. 1b). Hepatocytes in co-culture with HPc- and NPc-MSCs exhibited a similar morphology, with hepatocytes aggregated into large, oval-shaped colonies and attached on top of the MSCs in a three-dimensional manner, resulting in higher plating efficiency than mono-culture. HPc of MSCs potentiated their trophic and anti-apoptotic effects on hepatocytes with respect to albumin secretion on days 3–7 (*p* < 0.01; Fig. 1c), urea synthesis on days 3–7 (*p* < 0.01; Fig. 1d), caspase-mediated apoptosis on days 1–5 (soluble CCK18 release, *p* < 0.01; Fig. 1e), as well as total cell death on days 1–5 (soluble CK18 release, *p* < 0.01; Fig. 1f) without changing the death mode of hepatocytes (CCK18/CK18 ratio; data not shown). Indirect co-culture of hepatocytes with HPc- or NPc-MSCs in transwell plates and also culture with co-culture conditioned medium in order to test the involvement of soluble factors due to paracrine mechanisms showed minimal hepatotrophic and anti-apoptotic effects (data not shown).

Exposure of mono-cultured hepatocytes to 1 μM upwards of staurosporine significantly increased caspase-mediated apoptosis and total cellular death of hepatocytes and switched hepatocyte death mode from necrosis to apoptosis (all *p* < 0.01; Fig. 1g and h), as expected from this well-described apoptosis inducer. Co-culture with HPc-MSCs inhibited staurosporine-induced hepatocyte apoptosis to a greater extent than NPc-MSCs (*p* < 0.01; Fig. 1g) but had no further effect on staurosporine-induced total cellular death (Fig. 1h). Co-culture with HPc- and NPc-MSCs reversed the staurosporine-induced necrosis-to-apoptosis switch in hepatocyte death mode to a similar extent.

Pretreatment of MSCs with 5 mM NAC did not completely eliminate the HPc-induced increase in ROS activity in MSCs; however, the addition of 10 or 20 mM NAC prevented the HPc-induced ROS activity increase (both *p* < 0.01; Fig. 1a and b), with levels lower than those observed in NPc. Treatment of MSCs with 10 mM NAC significantly diminished the effects of HPc-MSC co-culture on hepatocyte albumin secretion on days 1–7 (*p* < 0.01; Fig. 1c) and urea synthesis days 3–7 (*p* < 0.01; Fig. 1d). It also reversed the effects on caspase-mediated apoptosis on days 1–5 (*p* < 0.01; Fig. 1e, upper panel) as well as total cellular death on days 3–5 (*p* < 0.01; Fig. 1f). The death mode of co-cultured hepatocytes switched from necrosis to apoptosis only on day 1 (data not shown).

### HPc inhibits TNF-α secretion of co-cultured hepatocytes but potentiates TGF-β1 activities of MSCs and co-cultured cells

**Fig. 2:**
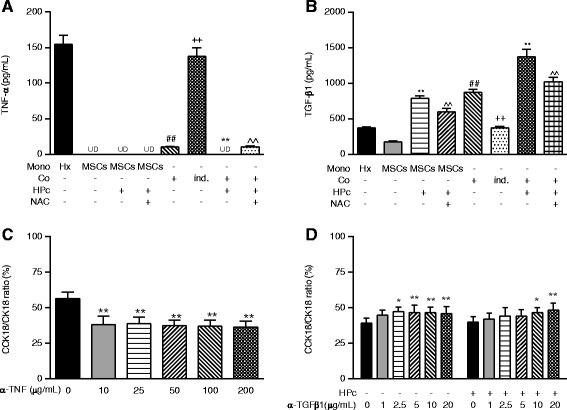
Decreased TNF-α and increased TGF-β1 in hepatocytes co-cultured with MSCs related to apoptosis. **a** TNF-α secretion of mono-/co-cultured hepatocytes and NPc-/HPc-MSCs. **b** TGF-β1 secretion of mono-/co-cultured hepatocytes and NPc-/HPc-MSCs. **c** Neutralisation with anti-TNF inhibits cellular apoptosis and, to a lesser extent, total death of mono-cultured hepatocytes, switching the main cell death mode from apoptosis to necrosis. **d** Neutralisation of MSCs with anti-TGF-β1 induces cellular apoptosis and, to a lesser extent, total death of co-cultured hepatocytes, switching the main cell death mode from necrosis to apoptosis. Values are mean ± standard deviation (*n* = 6). *p < 0.05 and **p <0.01, versus control mono- or co-culture; ^^*p* <0.01, versus co-culture with HPc-MSCs; ^##^ *p* <0.01, versus control mono-culture; ^++^ *p* <0.01, versus co-culture. *α-TGF-β1* Anti-transforming growth factor-β1, *α-TNF* Anti-tumour necrosis factor, *CCK18* Caspase-cleaved cytokeratin 18, *CK18* Cytokeratin 18, *Co* Co-culture, *HPc* Hypoxia-preconditioned, *Hx* Hepatocyte mono-culture, *ind.* Indirect, *Mono* mono-culture, *MSC* Mesenchymal stem cell, *NAC* N-acetylcysteine, *NPc* Normoxia-preconditioned, *UD* undetectable

Mono-cultured hepatocytes secreted a high level of TNF-α, while NPc-, HPc-, and NAC-pretreated HPc-MSCs secreted no detectable TNF-α (Fig. 2a). Co-culture with NPc-MSCs reduced hepatocyte secretion of TNF-α (*p* < 0.01), while indirect co-culture had no effect on TNF-α production (*p* < 0.01). Co-culture with HPc-MSCs decreased TNF-α secretion to a greater extent than with NPc-MSCs (*p* < 0.01), while ROS antagonisation with NAC to a smaller extent prevented the effects seen in HPc-MSC co-cultures (*p* < 0.01; Fig. 2a). TNF-α neutralisation with antibody significantly inhibited caspase-mediated apoptosis of mono-cultured hepatocytes from the lowest dose used (10 μg/mL; *p* < 0.01; Additional file 1: Figure S2A). TNF-α neutralisation at 25 μg/mL also significantly suppressed total death of hepatocytes (*p* < 0.01; Additional file 1: Figure S2C). This resulted in a switch in the main death mode of mono-cultured hepatocytes from apoptosis to necrosis (*p* < 0.01; Fig. 2c).

Mono-cultured hepatocytes and MSCs secreted high levels of TGF-β1 (Fig. 2b). HPc significantly increased MSC secretion of TGF-β1 (*p* < 0.01), which was reversed by ROS antagonisation with NAC (*p* < 0.01, Fig. 2b). Co-culture significantly increased TGF-β1 secretion as compared to hepatocyte or MSC mono-culture (both *p* < 0.01), while indirect co-culture abolished these effects (*p* <0.01 versus co-culture). Co-culture with HPc-MSCs further increased TGF-β1 secretion as compared to NPc-MSC co-culture (*p* <0.01), while this was partly reversed by NAC treatment (*p* < 0.01; Fig. 2b). Neutralising MSC autocrine activity of TGF-β1, starting from the 5 μg/mL dose, significantly diminished anti-apoptotic (*p* <0.01; Additional file 1: Figure S2C) and pro-survival effects (*p* <0.01; Additional file 1: Figure S2D) of co-culture with NPc- and HPc-MSCs. TGF-β1 neutralisation by antibody accompanied a necrosis-to-apoptosis switch in death mode of hepatocytes co-cultured with NPc-MSCs starting from 2.5 μg/mL and with HPc-MSCs from 10 μg/mL (*p* < 0.01; Fig. 2d).

### Effect of HPc-induced MSC co-culture with hepatocytes is related to deposition of extracellular collagen

**Fig. 3:**
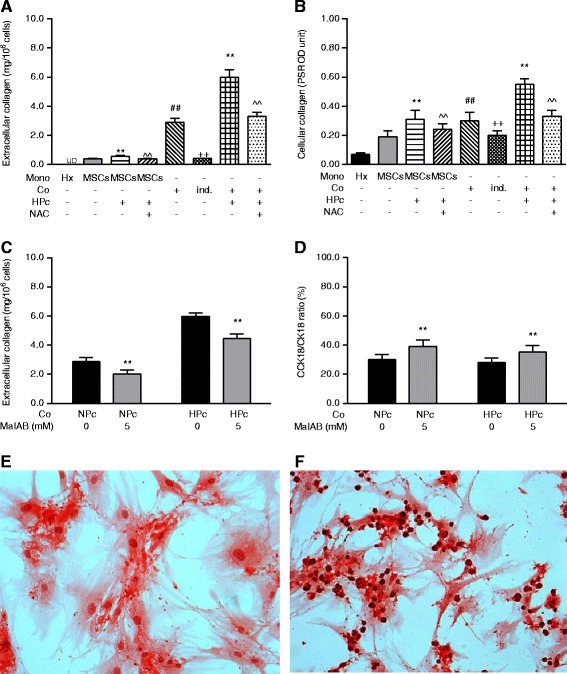
Dependence of co-culture hepatotrophic and HPc-induced potentiative effects on ROS-dependent MSC deposition of extracellular collagen. **a** Extracellular and **b** cellular collagen contents of mono-/co-cultured hepatocytes and NPc-/HPc-MSCs with or without NAC addition. **c** Inhibitory effect of MaIBA on extracellular collagen deposit of NPc- and HPc-MSCs. **d** Effects of 5 mM MaIBA pretreatment on main cellular death mode of hepatocytes co-cultured with NPc- versus HPc-MSCs. **e** and **f** Picro-sirius red staining (200×) for cellular and extracellular collagen of MSCs in mono-culture (*left panel*) and co-culture (*right panel*); nuclei are counterstained with hematoxylin. Values are mean ± standard deviation (*n* = 6). *p <0.05 and **p <0.01, versus control mono- or co-culture; ^^*p* <0.01, versus co-culture with HPc-MSCs; ^##^ *p* <0.01, versus control mono-culture; ^++^ *p* <0.01, versus co-culture. *CCK18* Caspase-cleaved cytokeratin 18, *CK18* Cytokeratin 18, *Co* Co-culture, *HPc* Hypoxia-preconditioned, *Hx* Hepatocyte mono-culture, *ind.* Indirect, *MaIBA* N-(methylamino)-isobutyric acid, *Mono* mono-culture, *MSC* Mesenchymal stem cell, *NAC* N-acetylcysteine, *NPc* Normoxia-preconditioned, *PSR OD Picro-sirius red OD reading*, *UD* undetectable

MSCs secreted large amounts of both cellular and extracellular collagen (Fig. 3a, b and e). HPc increased MSC deposition of cellular and extracellular collagen by intracellular ROS-dependent mechanisms (both *p* < 0.01). Co-culture significantly increased overall cellular and extracellular collagen production as compared to hepatocyte or MSC mono-culture (both *p* < 0.01; Fig. 3a, b and f). Co-culture with HPc-MSCs further increased cellular and extracellular collagen production as compared to that with NPc-MSCs (both *p* < 0.01), and this was significantly decreased by NAC-induced ROS antagonisation (both *p* < 0.01). Indirect co-culture resulted in an increased extracellular collagen deposition from hepatocytes (*p* < 0.01; Fig. 3b), though this was lower than in either NPc or HPc co-cultures with MSCs.

N-(methylamino)-isobutyric acid pretreatment to inhibit collagen production at the predefined concentrations used had no cytotoxic effects on MSCs as evidenced by MTT measurement and sulforhodamine B attachment assays (data not shown). Pretreatment starting from 5 mM significantly inhibited extracellular collagen deposition of NPc-MSCs (*p* <0.01; Additional file 2: Figure S3A) and HPc-MSCs (*p* < 0.01; Additional file 2: Figure S3B). Pretreatment of MSCs with 5 mM N-(methylamino)-isobutyric acid significantly inhibited extracellular collagen deposition of co-culture with NPc- and HPc-MSCs (both *p* < 0.01; Fig. 3c). Pretreatment also significantly diminished the anti-apoptotic effect of co-culture and HPc-induced potentiation (both *p* < 0.01; Additional file 2: Figure S3C) but had no significant effects on total cellular death of hepatocytes co-cultured with NPc- and HPc-MSCs (Additional file 2: Figure S3D). Pretreatment resulted in a necrosis-to-apoptosis switch of main death mode in hepatocytes co-cultured with NPc- and HPc-MSCs (both *p* < 0.01; Fig. 3d).

### HPc-MSCs downregulate expression of pro-apoptotic mRNAs by intracellular ROS-dependent mechanisms

**Fig. 4:**
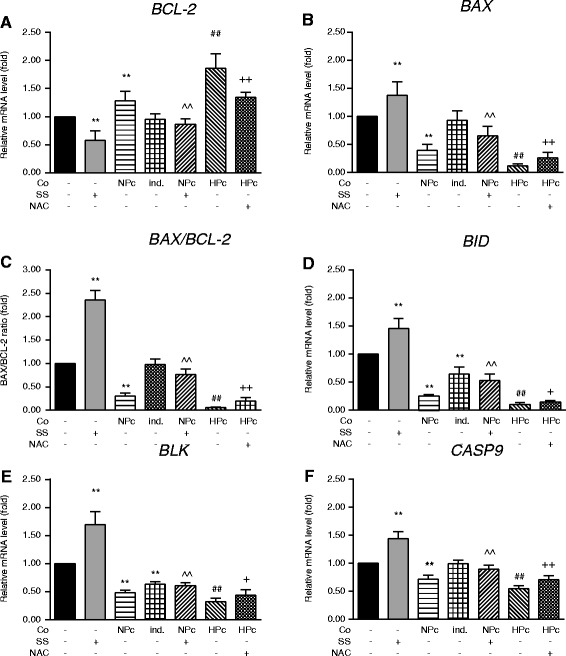
Relative mRNA expression levels of pro- and anti-apoptosis-associated genes in mono- and co-cultured hepatocytes. **a** *BCL-2*, **b** *BAX*, **c** *BAX*/*BCL-2* ratio, **d** *BID*, **e** *BLK* and **f** *CASP9*. Values are mean ± standard deviation (*n* = 6). **p <0.01, versus hepatocyte mono-culture; ^§§^ *p* <0.01, versus co-culture; ^^^^ *p* <0.01, versus SS-treated, mono-cultured hepatocytes; ^##^ *p* < 0.01, versus co-culture with NPc-MSCs; ^+^ *p* < 0.05 and ^++^ *p* < 0.01, versus co-culture with HPc-MSCs. *BAX* Bcl-2-associated X protein, *BCL-2* B-cell lymphoma 2, *BID* BH3 interacting-domain death agonist, *BLK* B lymphoid tyrosine kinase, *CASP* Caspase, *Co* Co-culture, *HPc* Hypoxia-preconditioned, *ind.* Indirect, *NAC* N-acetylcysteine, *NPc* Normoxia-preconditioned, *SS* Staurosporine

The mRNA expression of the anti-apoptotic *BCL-2* (Fig. 4a) was significantly upregulated in co-culture (*p* < 0.01) but not in indirect co-culture. The effect on *BCL-2* mRNA expression was potentiated when MSCs had been preconditioned with hypoxia (*p* < 0.01) and was diminished by addition of NAC (*p* < 0.01). At the same time, mRNA expression of pro-apoptotic *BAX* (Fig. 4b), *BID* (Fig. 4d), *BLK* (Fig. 4e) and *CASP9* genes (Fig. 4f) were all significantly downregulated in co-culture (*p* < 0.01). Indirect co-culture resulted in downregulation of *BID* and *BLK* mRNA expression (both *p* < 0.01), without any significant change in the expression of *BAX* or *CASP9*. HPc-MSC co-culture strongly downregulated the expressions of all pro-apoptotic genes (all *p* < 0.01), and upregulated the expression of *BCL2*, an effect that was significantly reduced by ROS antagonisation with NAC. The *BAX*/*BCL-2* ratio, used to assess cell drive towards apoptosis, was significantly decreased in co-culture (*p* < 0.01, Fig. 4c), but unchanged in indirect co-culture. This decrease was larger in co-culture with HPc-MSCs (*p* < 0.01) and diminished by ROS antagonisation (*p* < 0.01). Pretreatment with staurosporine, the inducer of apoptosis, upregulated mRNA expression of all apoptosis-related genes—*CASP9*, *BAX*, *BID* and *BLK* (all *p* < 0.01)—and downregulated that of *BCL-2* (*p* < 0.01) with a significant increase in *BAX*/*BCL-2* ratio (*p* < 0.01) in mono-cultured hepatocytes. However, co-culture prevented the staurosporine-induced upregulation of apoptosis-related gene mRNA expression (all *p* < 0.01) and downregulation of *BCL-2* mRNA expression (*p* < 0.01) resulting in a significant decrease in *BAX*/*BCL-2* ratio (*p* < 0.01). Expression of *CASP3*, *CASP8*, and *CASP14* mRNA was undetectable in mono- or co-cultured hepatocytes (data not shown).

## Discussion

This study has shown that hypoxia of MSCs significantly potentiates their effects on hepatocyte survival and metabolic function as reported previously in the presence of normoxia [8]. The mechanisms of these effects of MSCs have been investigated, supporting synergistic mechanisms involving soluble factors, ECM [19], and cell–cell crosstalk [20]. However, in our indirect co-culture experiments, soluble factors and paracrine mechanisms appeared to contribute minimally to the trophic effects of co-culture in NPc- and HPc-MSCs. Whilst it is possible that isolated primary human hepatocytes become unresponsive to soluble trophic and anti-apoptotic factors released from MSCs, our results suggest that the hepatotrophic effect of co-culture and HPc-induced potentiative effects are primarily mediated by synergistic effects of ECM and direct cell–cell interaction.

Intracellular ROS are known to modulate the biological activities of MSCs, including cellular proliferation, survival/apoptosis, and attachment. Exposure to moderate hypoxia is known to immediately increase intracellular ROS production in human MSCs [21], while pre-treatment with NAC, as used in this study, significantly inhibits the greater ROS production and associated cellular activities in hypoxic MSCs [22]. Our results showed that HPc potentiated the hepatotrophic effects of MSCs on co-cultured hepatocytes and that ROS antagonisation with NAC diminished these HPc-induced potentiative effects. Induction of ROS production accompanies phosphorylation of epidermal growth factor receptor, antagonisable by NAC treatment in human MSCs [23]. Aged adipose tissue-derived MSCs have a relatively low angiogenic capacity associated with downregulation of VEGF, placental growth factor, and hepatocyte growth factor expression, but HPc significantly upregulated expression of these pro-angiogenic factors and restored the angiogenesis of aged adipose tissue MSCs [10]. De Barros et al. [24] also reported that HPc could improve angiogenic capacity of aged human adipose tissue-derived MSCs. It remains to be investigated whether ROS also regulate cell adhesion via the ECM in the trophic and anti-apoptotic effects of MSCs on co-cultured hepatocytes. Chemically induced oxidative stress was reported to upregulate expression of bone morphogenetic protein 2 and fibroblast growth factor 2 in human adipose tissue MSCs in an intra-MSC ROS-dependent manner [25]. These two cytokines are well known to actively participate in ECM formation and modification of bone and cartilage [26].

TNF-α has a bidirectional regulatory effect on both hepatocyte proliferation and apoptosis which is modulated by extrinsic and/or intrinsic factors. Co-culture with MSCs protected hepatocytes from apoptosis induced by high-levels of TNF-α [27]. TNF-α neutralisation significantly decreased spontaneous apoptosis and total death of mono-cultured hepatocytes, while co-culture with MSCs particularly after HPc significantly inhibited hepatocyte autocrine activity of TNF-α. The inhibitory effect of co-culture on hepatocyte autocrine TNF-α activity was dependent on MSC–hepatocyte contact and MSC intracellular ROS activity. Trophic and protective effects of MSCs on co-cultured hepatocytes might result from reduced pro-apoptotic TNF-α together with delivery of trophic factors. However, as already suggested by the partial effect of TNF-α antagonism, the decrease in TNF-α expression is not sufficient to explain the co-culture effects on hepatocyte viability. Indeed, the conditioned medium from MSC/hepatocyte coculture contained a low level of TNF-α and did not mimic direct co-culture, therefore suggesting the role of synergistic factors other than TNF-α.

Co-culture of hepatocytes with MSCs exhibited an additive or synergistic, contact-dependent effect on TGF-β1 production. Autocrine TGF-β1 activity of MSCs was required for co-culture trophic effects and HPc-induced potentiation as evidenced by neutralisation of MSC-derived TGF-β1. A previous study showed that functional enhancement in hepatocytes co-cultured with NIH/3 T3 cells could be eliminated by TGF-β1 depletion and restored by TGF-β1 reconstitution [28]. We have shown that hypoxia increases MSC secretion of TGF-β1 as in other studies [29] in a ROS-dependent manner for both mono- and co-culture. However, TGF-β1 released by MSCs might not act on hepatocytes through a paracrine mechanism as co-culture conditioned medium containing high-levels of TGF-β1 had no significant effects on cell function or survival.

MSC deposition of extracellular collagen was enhanced by co-culture with hepatocytes, being greatest with MSCs cultured under hypoxic conditions compared to normoxia and this, at least partially, contributed to trophic and anti-apoptotic effects of MSCs on co-cultured hepatocytes. Attachment of extracellular collagen facilitates the entry of hepatocytes into S-phase [30] and mediates aggregation of hepatocytes and intercellular contact [31]. Extracellular collagen was mainly located around MSCs, and inhibition of collagen type I/V production in MSCs significantly reduced hepatocyte synthesis of albumin and urea [5]. Other groups have shown that HPc significantly upregulated expression of collagen I, II, and X along with some other genes encoding ECM [32], accompanied by enhanced autocrine activity of TGF-β [33]. Enhanced MSC deposition of extracellular collagen might primarily result from MSC–hepatocyte contact and HPc-induced potentiation which was also related to intracellular MSC ROS activity.

Staurosporine is known to activate caspase-3 signalling, independently of caspases-8, -9, and -12 [34]. MSCs, particularly after hypoxia, suppressed staurosporine-induced apoptosis rather than necrosis of co-cultured hepatocytes. Cleavage of CK18 at the position detected by the M30 kit is considered to be initiated by caspase-9 and executed by caspases-3 and -7 [35]. Caspase-9 is the initiator of caspase-mediated apoptosis through the mitochondrial pathway regulated by c-Jun N-terminal kinase/stress-activated protein kinase signalling pathways [36]. Co-culture of hepatocytes with MSCs after HPc significantly downregulated expression of the caspase-9 gene rather than caspases-3, -8, and -14, suggesting that the anti-apoptotic effects of co-culture and HPc-induced potentiation mainly acted on initiation of apoptosis. This is consistent with the reduced cleavage of hepatocyte CK18 into CCK18 initiated by caspase-9. Bax/Bcl-2 balance regulates hepatocyte apoptosis [37]. BID interacts with Bax and mediates caspase-3 and -8 in apoptotic hepatocytes [38]. Co-culture with MSCs upregulated expression of Bcl-xL and Bcl-2 in islet cells [39], and MSC transplants alleviated ischaemia/reperfusion-induced hepatocyte apoptosis by upregulating Bcl-2 [40]. Co-culture with MSCs significantly decreased *BAX*/*BCL-2* ratio and *BID* expression in a cell contact-, MSC ROS activity-dependent manner. The biological role of BLK remains to be investigated in hepatocytes. Staurosporine significantly upregulated expression of BLK, while co-culture with MSCs prevented this effect in a cell contact-dependent manner. BLK might be involved in apoptosis and the inflammatory response of hepatocytes as BLK participates in pre-B-cell receptor-mediated activation of nuclear factor kappa B [41], which tunes the balance between apoptosis and proliferation of hepatocytes in response to TNF-α [42]. Indirect co-culture with MSCs downregulated expression of BID and BLK only, which might explain the minimal trophic and anti-apoptotic effects of any paracrine factors.

Extracellular vesicles have been shown to mediate some of the effects of MSCs on cells [43] with anoxia preconditioning being shown to further enhance the effects of vesicles produced from MSCs [44]. In our study, effects due to extracellular vesicles are less likely as the effects found in our experiments are cell-contact dependant as the use of (i) indirect co-culture or (ii) conditioned medium from the MSCs only contributed a small part of the trophic effects of MSCs observed in this study. This is in contrast to other studies which showed that MSC conditioned medium ameliorates cell damage in models of liver injury. Whether this is due to the type of MSCs or the specific culture conditions used in this study remains to be investigated.

## Conclusions

In conclusion, MSCs have trophic, anti-apoptotic, pro-survival, and protective effects on co-cultured hepatocytes, which are significantly enhanced by HPc. The HPc-potentiated effects of MSCs on hepatocytes in co-culture were due to heterotypic cellular interaction and intracellular ROS-dependent mechanisms while paracrine factors had a minimal effect due to the absence of ECM and intercellular crosstalk. Potential mechanistic factors consisted of decreased TNF-α expression in hepatocytes, increased MSC TGF-β1 expression, and enhanced extracellular collagen deposition by MSCs. It is likely that these factors may synergistically switch the balance away from apoptosis in hepatocytes. Yu et al. [45] recently reported that transplantation of HPc- rather than NPc-MSCs significantly enhanced liver regeneration and the survival of animals after a major hepatectomy. It is hoped that the bench-to-bedside translation of the present work using co-culture of HPc-MSCs and hepatocytes will increase the therapeutic efficacy of hepatocytes for use in hepatocyte transplantation.

## Additional files

Additional file 1: Figure S2. Effects of TNF-α and TGF-β1 antagonism on cell death pathways. (A) Apoptosis and (B) necrosis with TNF-α (C) apoptosis and (D) necrosis in hepatocytes. Values are mean ± SD (*n* = 6). **p* < 0.05 and ***p* < 0.01, versus control mono- or co-culture. (PDF 62 kb) Additional file 2: Figure S3. Effects of N-(methylamino)-isobutyric acid (MaIAB) on extracellular collagen deposition from MSCs and hepatocyte cell death. Titration of MaIAB and its effects on collagen deposition in (A) mono-culture of MSCs or (B) co-cultures. (C) Apoptosis and (D) necrosis assessment in NPc or HPc co-cultured cells. Values are mean ± SD (*n* = 6). **p* < 0.05 and ***p* < 0.01, versus control mono- or co-culture. (PDF 39 kb)

## Abbreviations

ATP: Adenosine triphosphate
BAX: Bcl-2-associated X protein
Bcl-2: B-cell lymphoma 2
Bcl-xL: B-cell lymphoma-extra large
BID: BH3 interacting-domain death agonist
BLK: B lymphoid tyrosine kinase
CASP: Caspases
CCK-18: Caspase-cleaved cytokeratin 18
cDNA: Complementary DNA
CK18: Cytokeratin 18
Ct: Cycle threshold
ECM: Extracellular matrix
ELISA: Enzyme-linked immunosorbent assay
ERK: Extracellular signal regulated kinase
HPc: Hypoxic preconditioning/hypoxic preconditioned
MAPK: Mutagens-activated protein kinase
MFI: Median fluorescence intensity
MSC: Mesenchymal stem cell
NAC: N-acetylcysteine
NPc: Normoxia-preconditioned
P: Passage
qRT-PCR: Quantitative real-time polymerase chain reaction
ROS: Reactive oxygen species
SD: Standard deviation
TGF: Transforming growth factor
TNF: Tumour necrosis factor
VEGF: Vascular endothelial growth factor
