# Supplementary figures and images for: Hypoxic preconditioning potentiates the trophic effects of mesenchymal stem cells on co-cultured human primary hepatocytes
Source: Stem Cell Res Ther. 2015 Dec 1;6:237. doi: 10.1186/s13287-015-0218-7 (PMC4667488; doi:10.1186/s13287-015-0218-7)

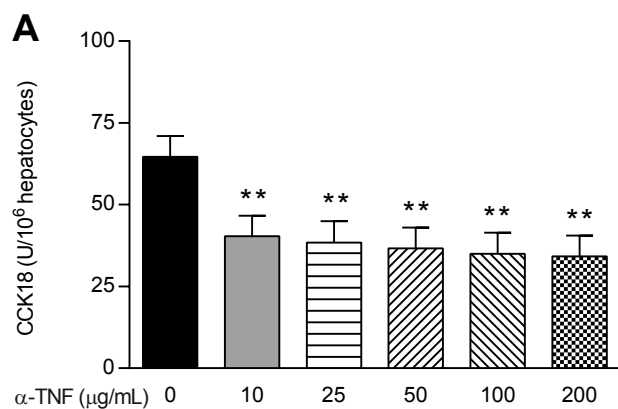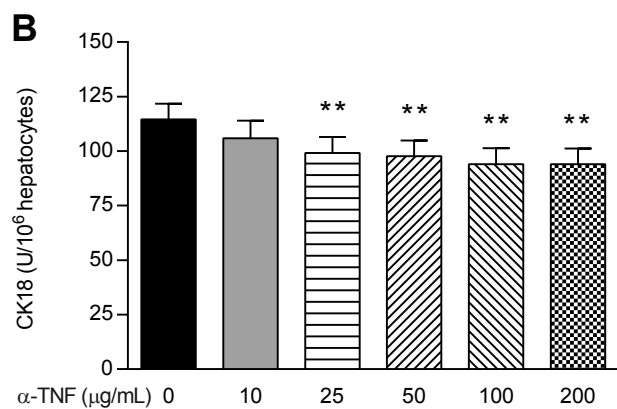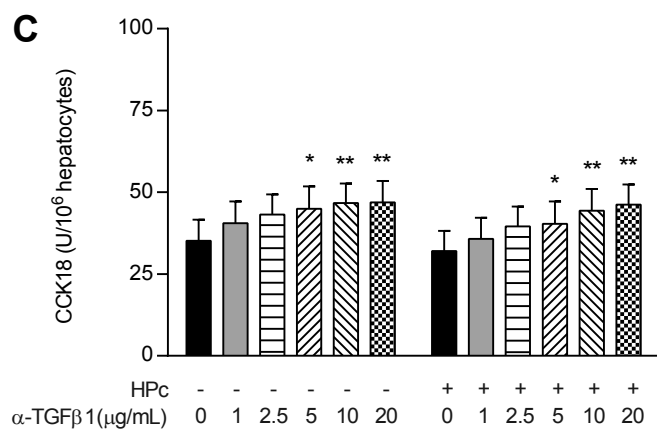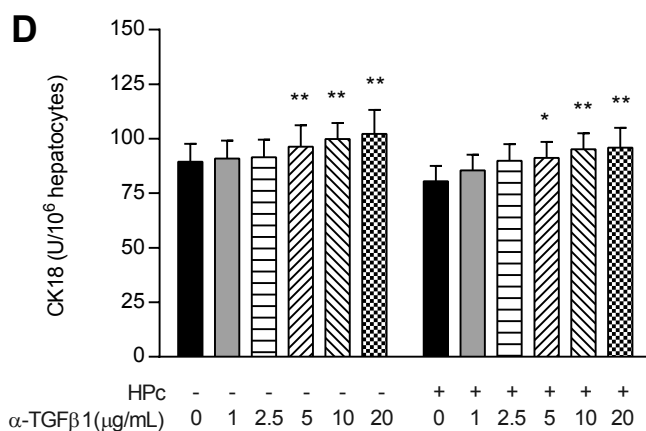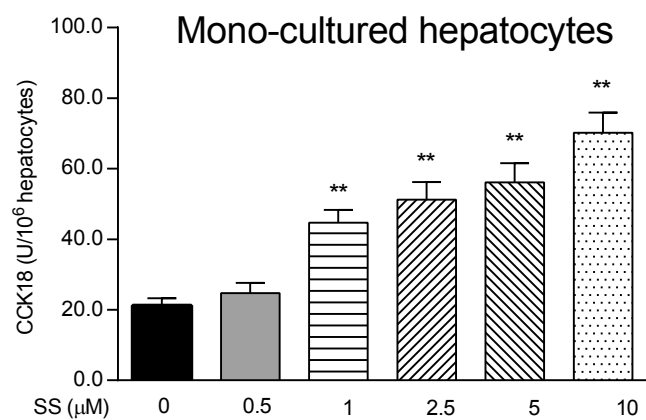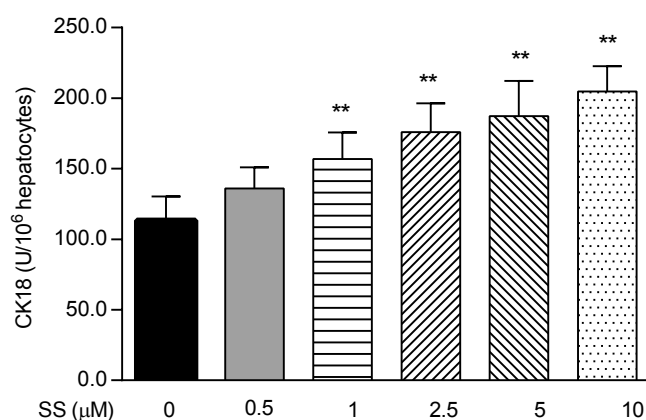

Supplement: Additional file 1: Figure S2. — Effects of TNF-α and TGF-β1 antagonism on cell death pathways. (A) Apoptosis and (B) necrosis with TNF-α (C) apoptosis and (D) necrosis in hepatocytes. Values are mean ± SD (n = 6). *p < 0.05 and **p < 0.01, versus control mono- or co-culture. (PDF 62 kb) [file 13287_2015_218_MOESM1_ESM.pdf]

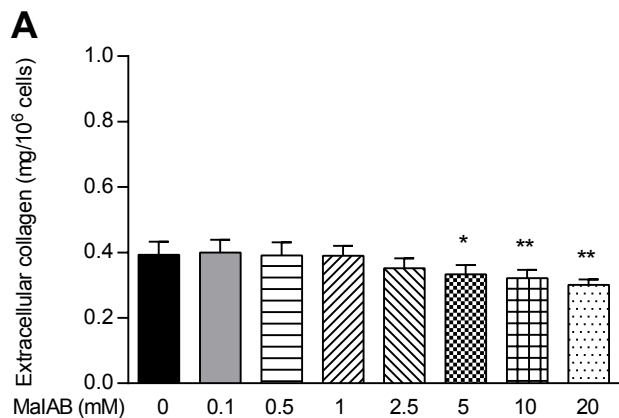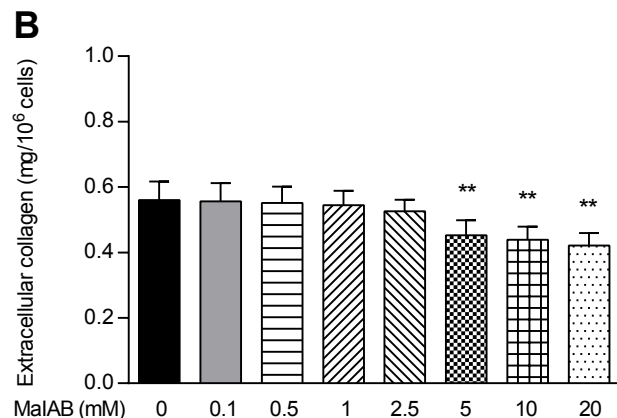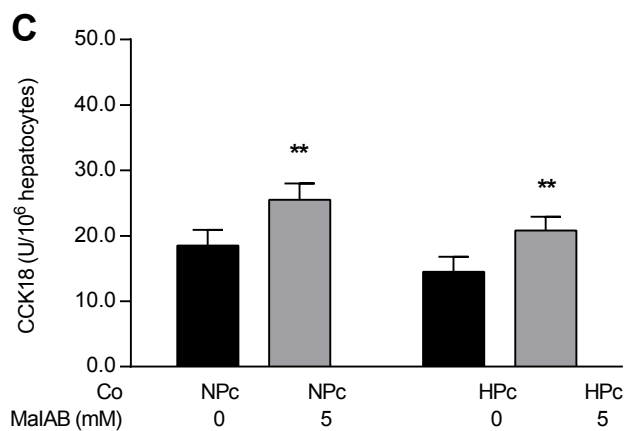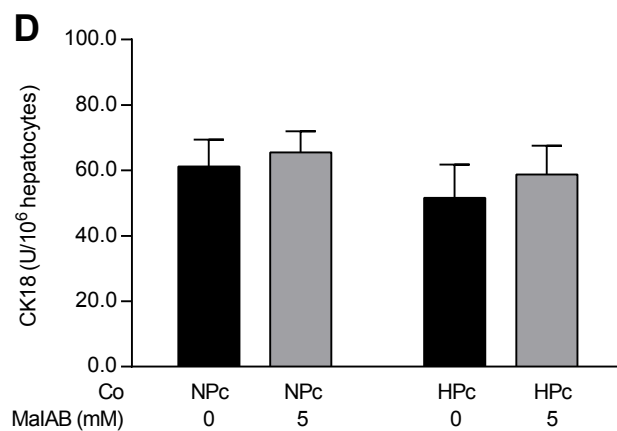

**Supp. fig. 3**

Supplement: Additional file 2: Figure S3. — Effects of N-(methylamino)-isobutyric acid (MaIAB) on extracellular collagen deposition from MSCs and hepatocyte cell death. Titration of MaIAB and its effects on collagen deposition in (A) mono-culture of MSCs or (B) co-cultures. (C) Apoptosis and (D) necrosis assessment in NPc or HPc co-cultured cells. Values are mean ± SD (n = 6). *p < 0.05 and **p < 0.01, versus control mono- or co-culture. (PDF 39 kb) [file 13287_2015_218_MOESM2_ESM.pdf]
